# Testing Protocol Development for the Fracture Toughness of Parts Built with Big Area Additive Manufacturing

**DOI:** 10.3390/polym16162321

**Published:** 2024-08-16

**Authors:** J. P. Garcia, L. A. Camacho, A. I. Villegas, A. Hasanyan, D. Espalin

**Affiliations:** 1 Department of Aerospace and Mechanical Engineering, The University of Texas at El Paso, 500 W. University Ave, El Paso, TX 79968, USAaivillegas3@miners.utep.edu (A.I.V.); adhasanyan@utep.edu (A.H.); 2 W. M. Keck Center for 3D Innovation, 500 W. University Ave, El Paso, TX 79968, USA

**Keywords:** large-scale additive manufacturing, double cantilever beam test, fracture toughness, digital image correlation, material extrusion

## Abstract

The mechanical testing of additively manufactured parts has largely relied on the existing standards developed for traditional manufacturing. While this approach leverages the investment made in current standards development, it inaccurately assumes that the mechanical response of additive manufacturing (AM) parts is identical to that of parts manufactured through traditional processes. When considering thermoplastic, material extrusion AM, the differences in response can be attributed to an AM part’s inherent inhomogeneity caused by porosity, interlayer zones, and surface texture. Additionally, the interlayer bonding of parts printed with large-scale AM is difficult to adequately assess, as much testing is performed such that stress is distributed across many layer interfaces; therefore, the lack of AM-specific standards to assess interlayer bonding is a significant research gap. To quantify interlayer bonding via fracture toughness, double cantilever beam (DCB) testing has been used for some AM materials, and DCB has been generally used for a variety of materials including metal, wood, and laminates. Mode I DCB testing was performed on thermoplastic matrix composites printed with Big Area Additive Manufacturing (BAAM). Of particular interest was the notch shape and deflection speed during testing. The results examine the differences when using two notch types and three deflection speeds. The testing method introduced by the following paper differentiates itself from the ones described in the standards used by modernizing the methodology. This was conducted with the introduction of Digital Image Correlation (DIC) to gather displacement and load data simultaneously without human intervention.

## 1. Introduction

Although additive manufacturing technology is advancing significantly, there remains a considerable lack of standards for characterizing the mechanical properties of additive manufacturing (AM) materials. It is important to mention that, though a lack of standards exists, there are efforts to compile standards that serve as the basis for testing AM materials. Kawalkar et al. [1] highlighted in their review both the need for standards in AM and the efforts made to compile them. The focus of this paper is the mechanical characterization of material extrusion (MEX) composites for large-scale additive manufacturing, specifically fracture toughness at the layer interface.

It is known that for traditionally manufactured composite materials with a polymer matrix, the materials are anisotropic in their mechanical properties, something also true for parts built with MEX. Important factors to be considered for the material characterization of composites are fiber orientation, fiber continuity, and stacking sequence. In the literature, it is established that in large-scale AM there is some alignment of the short fibers to the printing direction on the x/y plane at the layer interface [2,3,4]. For this project, it was assumed that the fiber orientation on the print was largely unidirectional to narrow the search for a standard to consult. It is relevant to recognize that after extrusion the material shrinks and deforms, which adds complexity to fiber orientation; this is called nesting [5], which is important to observe as it may impact the interaction at the layer interface. The stacking sequence for each layer is assumed to be at 0°, with the reference axis being the printing direction and fibers are either short or discontinuous at the layer interface of a print. Older studies looking at the impact of the introduction of short fibers in thermoplastics showed an increase in fracture toughness, although it is still a weak point of the composite [6]. These composites have a combination of good mechanical properties with overall better thermal and mechanical properties, making them attractive for usage in large-scale AM [7]. Comparing traditionally manufactured techniques with AM, the phenomenon that most closely adheres to failure at the layer interface is fiber bridging [8].

To test the fracture toughness of unidirectional fiber-reinforced polymer matrix composites, a double cantilever beam (DCB) mode I method based on ASTM D5528 [9] is used. For the project, this standard is used to describe layer interface behavior and for validation. The use of ASTM D5528 to characterize the layer interface for large-scale AM thermoplastic composites has precedent since Kishore et al. [10] used it to test the individual inter-layer strength and energy release of the crack to initiation. The standard recommends the stain rate to be between 1 and 5 mm/s, which is relevant since in the literature it is suggested that different strain rates impact the average fracture toughness of the material [11]. Thus, testing at different strain rates and performing a statistical analysis to find consequential differences in the results is part of the scope of this investigation.

Additionally, ASTM D5528 recommends using a microscope or any other observation tool that can help visually detect, with a precision of ±0.5 mm or less, the crack length and then record these measurements to then use in calculations. The novel approach of the project lies in instead implementing digital image correlation, a feature already implemented in testing large-scale AM materials. An example being the work conducted by Schnittker et al. [12], who used digital image correlation on a test based on ASTM D638 [13], a standard also consulted for this project.

The examination of fracture toughness in additive manufacturing (AM), particularly in large-scale AM, is limited in scope. Research related to the fracture toughness in AM is focused on characterizing parts fabricated by regular scale MEX [14,15,16]. An example is Papon and Haque [15], who tested the fracture toughness of 3D-printed composites using a mode I compact specimen test, recognizing a research gap but focusing on the effects of nozzle geometry on fracture energy. Additionally, fracture toughness at the layer interface is not relevant for a lot of these research efforts, although advancements in research have been made. Young et al. [17] followed ASTM D5528 to characterize the interlayer fracture toughness of a 3D-printed part without adjusting the standard. This is not possible to do in large-scale AM due to the limitations attached to the scale of the print. This investigation specifically focuses on characterizing fracture toughness at the interlayer level for parts fabricated using large-scale AM.

The fracture toughness at the layer interface serves as an indicator of the strength of bonds between layers. By shedding light on the material’s capabilities and assessing its controllability in this aspect, it enables further customization for specific applications. Moreover, it establishes a foundation for future standardization efforts in additive manufacturing, encompassing specialized specimen geometries and strain rates. In addition to bridging existing knowledge gaps, this paper introduces a pioneering approach to recording fracture toughness data by using digital image correlation (DIC). A MATLAB script was developed to process both video and test data post-experiment, enhancing the analysis process of crack propagation [18] by automating the previously manual measurements suggested in ASTM D5528. The study also uses statistical analysis to assess the suggestions made by the standard concerning the use of different strain rates and preload to set a precedent for further implementation of the method proposed.

## 2. Methodology

**Figure 1:**
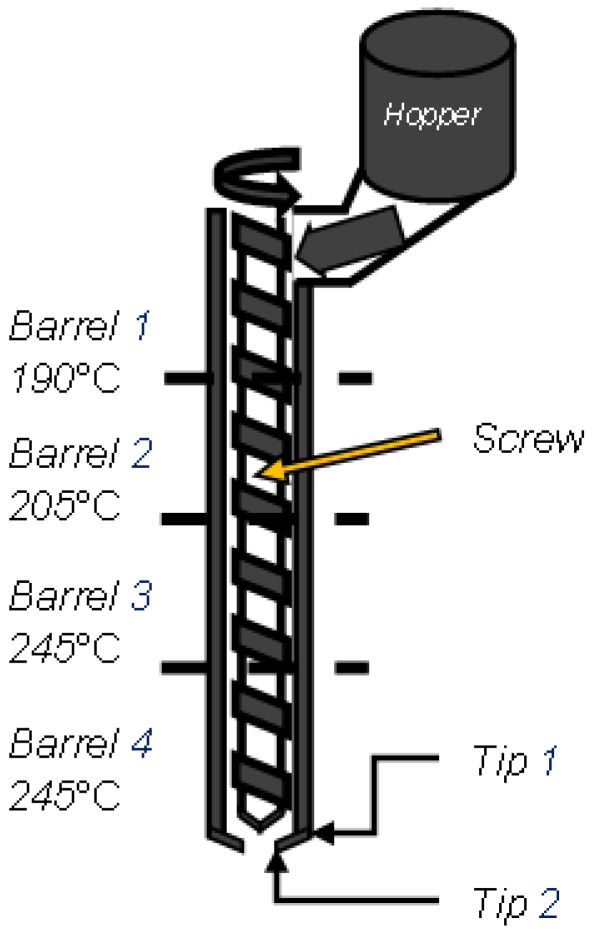
Heated zones and hopper system for the extruder.

For the experiment, the material used was Electrafil^®^ ABS 1501 3DP (Techmer PM, Wichita, KS, USA), which was 20 wt.% carbon-fiber-filled acrylonitrile butadiene styrene (ABS). The CF-ABS was dried for four hours at 90 °C for extrusion. The material was extruded at 245 °C while being heated continuously inside the barrels from 190, 205, 245, and 245 °C, respectively (Figure 1). The geometry used for the harvesting of coupons was a single-wall hexagon [12] with a wall length of 304.8 mm and a height of 217.51 mm. The slicer used was Oakridge National Lab’s Slicer 2 (Oak Ridge National Labs, Oak Ridge, TN, USA), with a layer height of 3.5 mm. A hexagonal shape was chosen [5], so the six panels were printed with minimal deformation and in similar printing conditions. Each panel in turn was designed to yield six coupons for an adequate number of testing samples. The samples were printed using a Big Area Additive Manufacturing (BAAM) machine developed by Cincinnati and Oakridge National Laboratory (ORNL).

**Figure 2:**
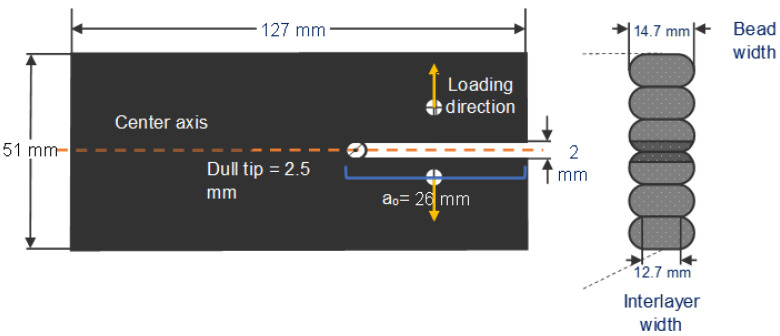
DCB sample geometry.

To harvest the coupons, the panels were cut from the hexagon using water jetting. Water jetting was chosen over a hacksaw or horizontal band saw because it is less thermally invasive to the coupon. The geometry of the sample (Figure 2) was selected following what Kishore et al. [10] used for interlayer bonding characterization. Due to the scale of the print, it was impossible to follow the dimensional ratios recommended by ASTM D5528. The length of the coupon was chosen due to the limitations of the testing rig (Instron 5866, Instron, Norwood, MA, USA), while the height was selected to prevent transversal cracking. The most critical geometry feature is the point at which the load is applied and the tip of the pre-crack, which is defined by a_0_ (Figure 2), the symmetry of the specimen along the fracture plane and interlayer width.

**Figure 3:**
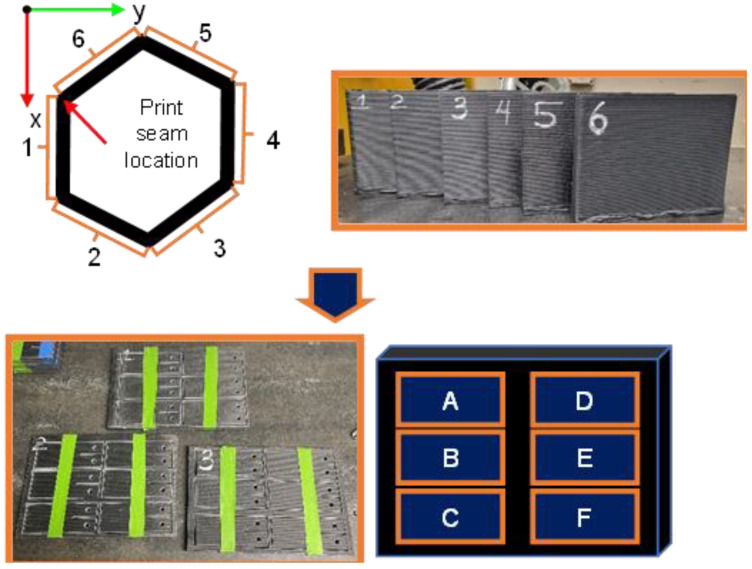
Specimen nomenclature and harvesting process diagram.

After the fabrication of each sample, they went through a labeling process. The coupons were labeled with a number from 1 to 6 corresponding to the face of the hexagon they belonged to. The numbers were accompanied by a letter from A to F in accordance with the positioning of the coupon within the panel (Figure 3). Additionally, for testing, the coupons were conditioned with the help of the reference of ASTM standard D618, which goes into the detail of conditioning procedures. It was stated on the standard to store and test at a laboratory atmosphere of 23 °C ± 3 °C in temperature and 50 ± 10% relative humidity.

**Figure 4:**
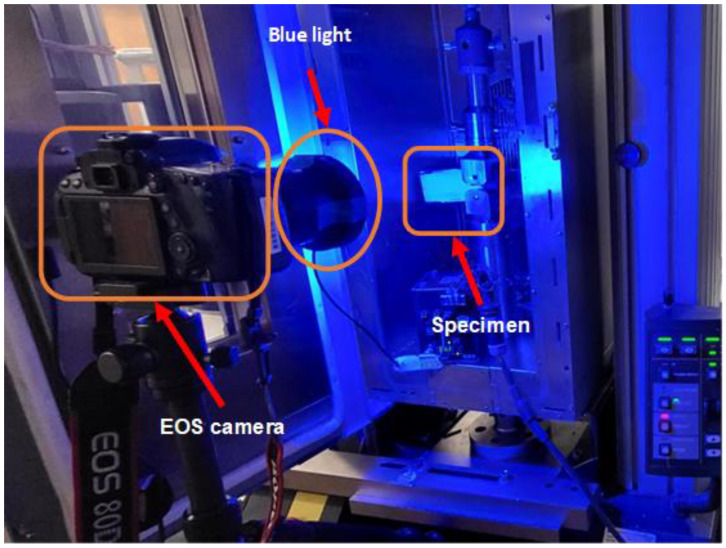
Experimental set up. The specimen was placed inside the Instron 5866, coated in white. In front of the Instron, the EOS camera is placed over a tripod with a blue light lamp over the lens providing contrast for the image processing.

The Instron 5866 was fitted with a 10 kN load cell for the testing, while Bluehill2 software (Instron, Norwood, MA, USA) (Figure 4) was used to record load and crosshead displacement. To correlate the load–displacement curves with the specimen’s specific crack length, synchronized video recording was used. The equipment selected for the video recording was a Canon EOS 80D camera equipped with a Canon EF 10 mm f/2.8 L Macro IS USM lens CA (Canon, Melville, NY, USA) (Figure 4). The information gathered from the Instron and the camera was used to calculate fracture toughness.

**Figure 5:**
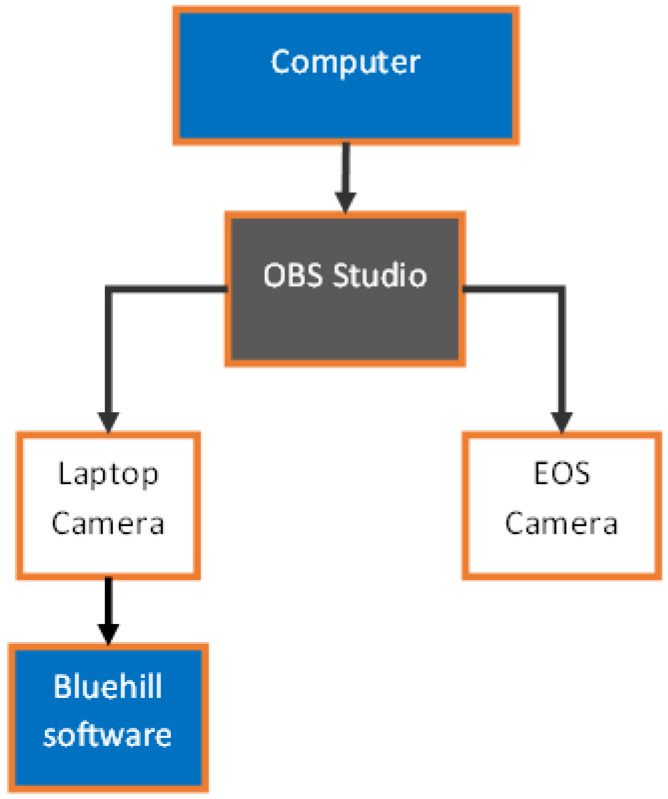
Data synchronization workflow diagram.

Prior to testing, the interlayer width was measured for every specimen using a caliper. After the measurement, a thin layer of white corrector fluid was applied as a coating for the specimens. A custom stencil was used to add the calibration marks on top of the coating. For the experiment, as load is applied to the specimen and the camera records the length crack propagation. A separate recording pointed at the Bluehill2 software is used so that both recordings can be synchronized and a relationship between the experimental load and displacement with the recorded crack length can be established using the frames of the recording. OBS studio (OBS 27.2) software was used to unify and synchronize both recordings (Figure 5). This technique allows gathering hundreds of points in the crack length. As mentioned before, the specimen surfaces were marked with calibration dots in two parallel rows that, in turn, were parallel to the pre-crack. Each row had eleven dots with a nominal distance of 5mm between them. It is of importance to mention that the stencil used for marking the dots was manufactured using vat photopolymerization, which carries dimensional inaccuracies. The dots were useful to correct distortion, to average the distance between adjacent dots, and for maintaining a consistent aspect ratio, as well as giving a general idea of the state of the image surrounding each dot.

The experimental set up consisted of the Canon EOS 80D camera being placed normal to the specimen’s crack propagation surface. A blue light was shined towards the white cover of the specimen for a higher contrast between the painted parts and the crack, background, and unpainted parts for image processing (Figure 4). Two batches of specimens were prepared in different ways; the first batch was tested with a small semicircle ending the pre-crack notch, while the second batch had an incision made with tungsten razorblades (DeWalt, Towson, MD, USA). The objective of the difference between pre-crack notches was to verify that by making the pre-crack notch ending thinner, instead of testing structural strength, the material properties would be tested. Apart from the specimens with a razor pre-crack or lacking it, six specimens were tested at speeds of 1, 3, and 5 mm/s with and without preloading.

**Figure 6:**
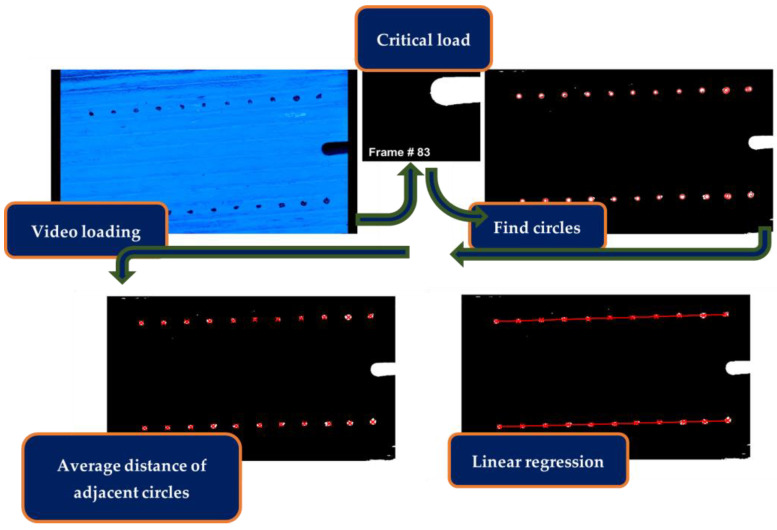
Calibration subsection workflow.

For DIC, MATLAB was used to extract frames and process them for correlation between frames and load–displacement and then record the data for each test (Figure 5). The MATLAB (2022b) process has a four-part workflow consisting of frame and data extraction, image calibration, crack propagation measurements, and fracture toughness calculations (Figure 6). The novelty of the process explained in this paper lies in frame extraction, calibration, and crack propagation, which will be explained in conjunction.

For the data acquisition, the test video was recorded at 60 frames per second, while the load–displacement data were recorded every 0.334 s. The timelapse for data acquisition was deliberate, since 0.334 s is a multiple of 60 frames per second. This is useful to make every load–displacement data point a frame to be extracted for the video of crack propagation. Both the crack propagation video and the screen recording from the Bluehill2 software recording load/displacement were merged into a single recording using OBS Studio for easy synchronization and frame extraction (Figure 5). The video was then trimmed, using a computer default editor to view, from the start of the testing to the end of the test. Per every load–displacement data point, a frame would be collected equidistantly in relation to time by MATLAB.

A crucial part of data acquisition was calibration. The intention of calibration was to size the pixels in terms of millimeters. The calibration method uses two rows of dots at the top and at the bottom of the specimen instead of a single gauge length (two dots) to find the pixel size ratio (Figure 6). Since the image resolution for every frame is the same, just one frame is needed for calibration. The frame selected for calibration was the one at which the specimen had reached critical load, thus named the critical frame. The critical frame was selected because, at this point of the testing, rotation from the initial loading reached stability and sat before crack propagation, where rotation from crack opening occurs (Figure 6). The workflow for calibration then consists of using the script to find the dots as circles and calculate the distance between the circle centers. These distances were intended to be 5mm from circle to circle, thus the number of pixels between each circle center was divided by this distance, giving a mm/pixel ratio. Since the set up might have changed from set up to set up, this method allowed for an undesirable tilt, which was compensated for by creating a linear regression per each row of dots (top and bottom) and using the average slope of both sides to compensate as the crack length propagated (Figure 6). The first few frames were neglected for the calculation since the crack length remained the same as the initial pre-crack, a_0_. Additionally, since the lens used on the camera for the testing was a narrow lens, distortion was negligible.

**Figure 7:**
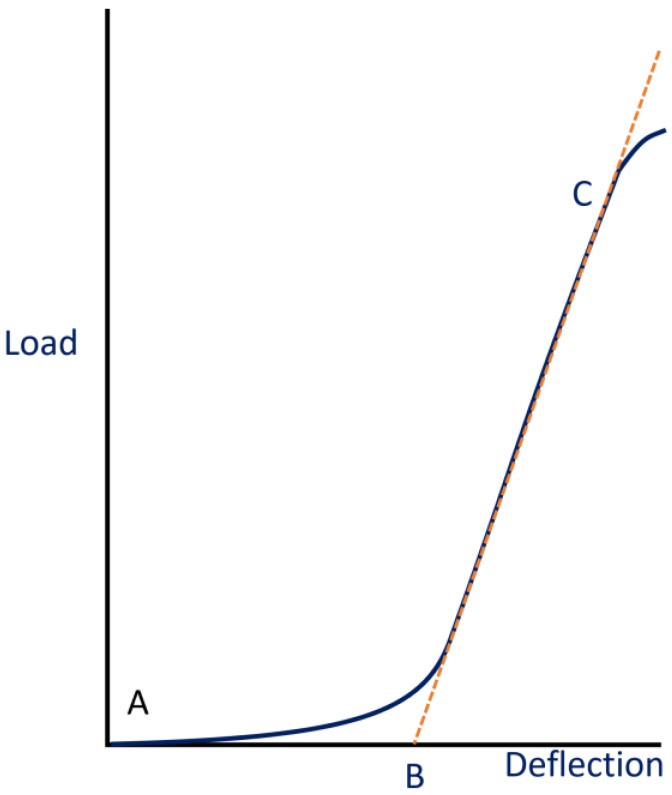
LvD offset correction method.

The formula used to calculate the fracture toughness (G_IC_) (Equation (1)) [19] was derived to accommodate for the shape of the specimens used in this project as well as the distribution and effects of the loading in the geometry. The plotted curve depicting load versus deflection (Figure 7), referred to as the corrected load vs displacement (LvD) plot in this study’s script, displayed certain characteristics. Prior to mounting the specimen, the load cell underwent calibration. However, upon mounting the specimen, there remained a small distance that needed to be covered before the load was applied. This additional deflection was deemed irrelevant to the testing process. Moreover, the plot exhibited a toe region, indicating progressive loading, which other standards such as ASTM D638 indicated was not indicative of the material’s behavior. It is worth noting that these correction methods are not specified in the DCB standard [8]. The decision to offset the values occurred when the linear region intersected the origin. This approach aimed to measure the distance at which loading onto the specimen commenced, rather than considering the deflection range where the opening did not impose a load on the specimen. In Figure 7, point A represents the test’s initiation, while line BC represents the regression of the LvD plot’s linear region. In this scenario, B denotes the new offset length, indicating the starting point of loading in the DCB test.

(1) $$G_{IC}=\frac{4P_{c}^{2}}{Eb^{2}h^{3}}[3{\left(a+a_{0}\right)}^{2}+h^{2}]$$

## 3. Results and Discussion

For the exploratory work, the testing was divided into two parts to qualify differences in testing techniques. These two testing blocks consist of preload versus no-preload and sharpened pre-crack versus unsharpened pre-crack, respectively. The goal of employing different testing techniques is to determine if these variations impact the testing. The theory behind sharpening the notch is that a dull pre-crack would test for structure instead of material properties.

**Figure 8:**
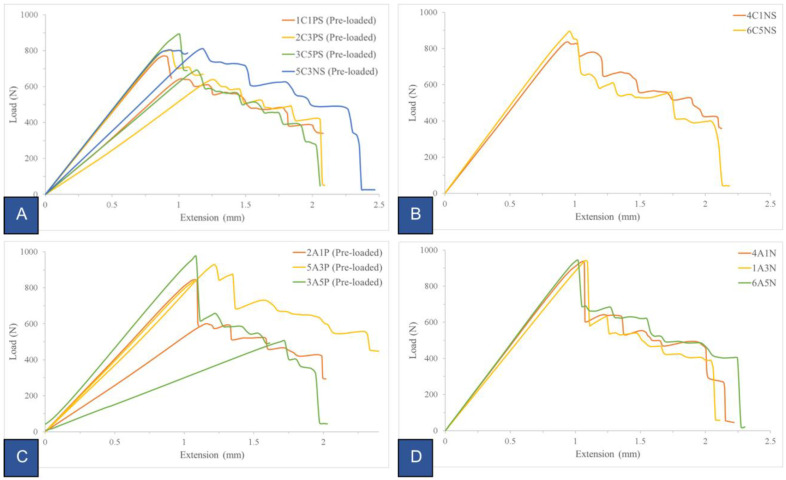
Load (N) versus Extension (mm) (crosshead deflection) curves. (**A**) Pre-loaded samples with notch sharpening; (**B**) no-pre-loaded samples with notch sharpening; (**C**) pre-loaded samples; (**D**) no-pre-loaded samples. Each line represents a different specimen tested in the same conditions.

During the initial round of tests (depicted in Figure 8C,D), an abrupt and nearly vertical behavior on the loading plot emerged right after reaching the critical load. This occurrence is referred to as an unstable brittle fracture. In the literature [20], it is clarified that such a situation is unfavorable for detecting fracture toughness. In contrast to the specimen with a sharpened notch, the specimen with a dull pre-crack can withstand a higher load (shown in Figure 8A,B). This dissimilarity arises not from a difference in material properties, but rather from structural factors. It is attributed to the lower stress concentration at the notch initiation point due to the geometry of the pre-crack tip. Furthermore, it is evident from the pre-loaded tests (illustrated in Figure 8A,C) that the control over the point where the pre-load was halted varied. This difference in behavior arose because the test had to be manually stopped since the testing software lacked an efficient control mechanism for determining the stopping point. Ideally, one should stop the test within a range of 3–5 mm of crack propagation before starting the second phase of the test. Considering that there is virtually no distinction between pre-load and no-pre-load conditions, it was recommended, for practical reasons, to opt for the no-preload approach during testing.

To obtain the results of DCB testing, each specimen generated two distinct graphs: a load versus deflection curve (LvD) and an R-plot. The R-plot illustrated the fracture toughness values at various points along the crack propagation length, including the filtered data from subsequent iterations. To ensure diverse results, the three testing parameters for deflection speed were randomized using Minitab. The tests were conducted without pre-load and with notch sharpening, based on the findings of the preliminary work. Fracture toughness calculations were performed using the Mostovoy et al., 1967 [19] method, referred to as the rigid specimen compliance calibration (RSCC) in this study. All the recorded data points from the critical load until the end of testing were utilized in the calculations. In other words, every load and deflection measurement taken after reaching the critical load was considered to analyze the behavior and intricacies of the testing. However, it is important to note that not all data points hold significance for fracture toughness calculations. To address this, the RSCC method was employed in a subsequent round of results analysis. This involved employing a discrimination criterion to select prominent peaks in the data, which were then used to calculate fracture toughness.

**Figure 9:**
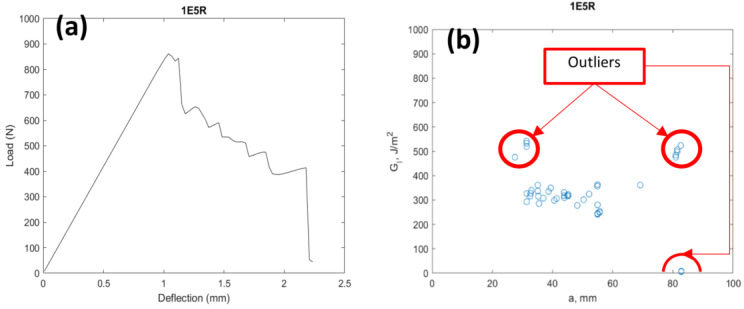
Unfiltered values of (**a**) LvD and (**b**) R-plot of specimen 1E5R.

**Figure 10:**
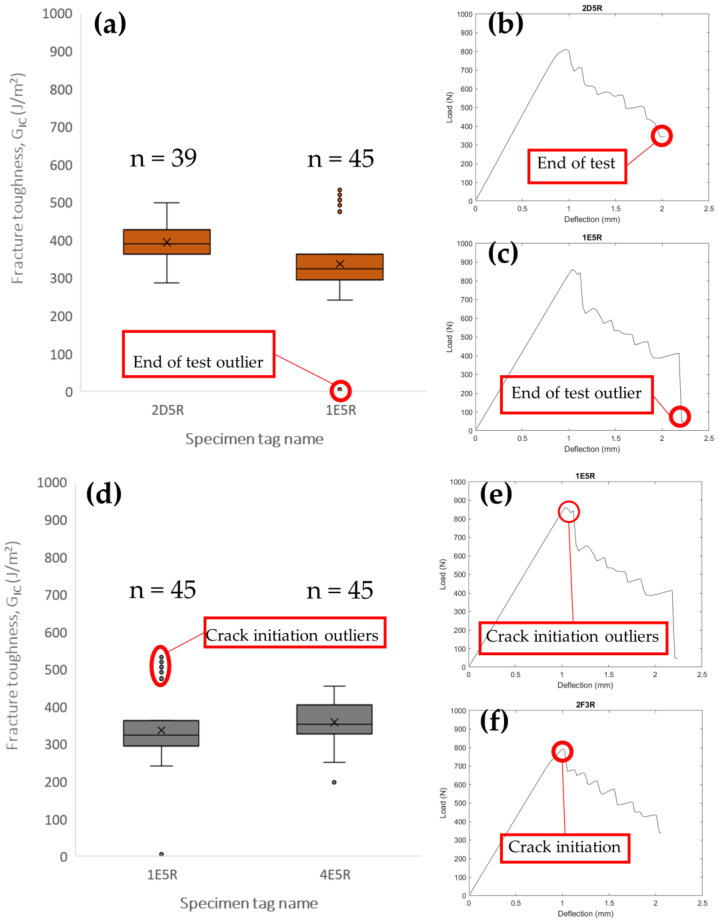
(**a**) Box plot unfiltered values comparison between (**b**) test stopped at the required crack length and (**c**) test stopped after critical failure. (**d**) Box plot unfiltered values comparison between (**e**) test of low flow impact crack sharpening and (**f**) high impact crack sharpening.

DCB mode I had a useful advantage for the current project, which is that from a single specimen, multiple G_IC_ values were yielded. This meant that each R-plot was the G_IC_ result for single specimens. The resolution of the points yielded by the Bluehill2 software is dependent on the deflection speed, that is why n values are specified for the plots. On the initial R-plots, or “unfiltered” R-plots as they will be referred to, the existence of outlier points is clearly visible (Figure 9). These points were classified as outliers since the G_IC_ value was considerably higher in comparison to the rest of the points. The positioning of the outlier points towards the start and the end of the R-plot indicates several causes for the high G_IC_ values (Figure 10). The cause for the outliers at the start of the plot was the dull pre-crack notch (Figure 10d–f). A dull pre-crack means that instead of testing for the material’s properties, the structural property of the pre-crack was being tested, leading to a higher fracture toughness; this phenomenon was also visible to an extent at some of the sharpened pre-crack specimens and, in a few, the G_IC_ was so high as to be indistinguishable from the tests where no sharpening was performed. The existence of such events reduced the pool of valid fracture toughness results on a case-to-case basis. As for the outliers at the end of the testing (Figure 10a–c), three non-mutually-exclusive events were theorized to cause it. First, a catastrophic fracture happened at the end of the testing, captured by the camera as a large and fast crack that tended to go out of frame. Since the testing method used in the current paper measures the crack propagation through visual processing, for a crack going out of frame when recording a high G_IC_ immediately after a catastrophic crack and no record of a length increase, the result was an abnormally low fracture toughness, resulting in an outlier. The third reason for outlier points was that, due to the speed of the crack propagation at catastrophic failure, the camera could not record the propagation, giving lower G_IC_ values. This indicated that the test should be stopped at a reduced crack length threshold compared to that indicated in ASTM D5528. Catastrophic failure is consistent with a crack length of 70 mm or a deflection point of 2 mm, this information can be used to determine the testing parameter relations related to the specimen’s length.

**Figure 11:**
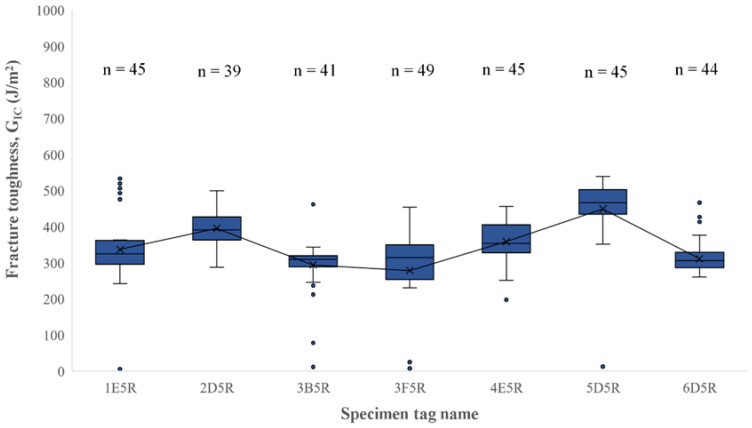
Unfiltered G_IC_ boxplot for fracture toughness using RSCC model.

The fracture toughness results were plotted with their corresponding deflection speeds into boxplots to better identify the outliers in the results (Figure 11). The threshold value was calculated by calculating the quartiles of the dataset. The quartiles were classified as Q_1_ and Q_3_ corresponding to the medians of the lower and upper halves of the dataset, respectively. For the outlier threshold, the Inter Quartile Range is calculated by obtaining the difference between Q_1_ and Q_3_. The upper and lower thresholds for the outliers are calculated by the following expression:

(2) $$Lower\;limit=Q_{1}-\left(IQR*1.5\right),\;\mathrm{and}$$

(3) $$Upper\;limit=Q_{3}+\left(IQR*1.5\right)$$

This is a preset to build the boxplots but can be modified if performed manually. Some important things noted on the testing were that since the test is dependent on time, higher deflection speeds yielded lower G_IC_ values. It is relevant to remember every specimen had sufficient fracture toughness values to construct a plot.

**Figure 12:**
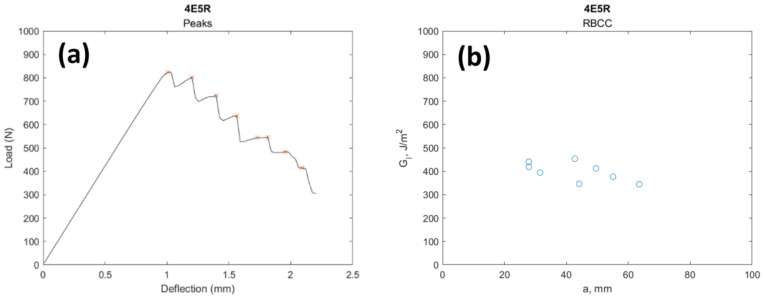
(**a**) R-plot and (**b**) peak values.

**Figure 13:**
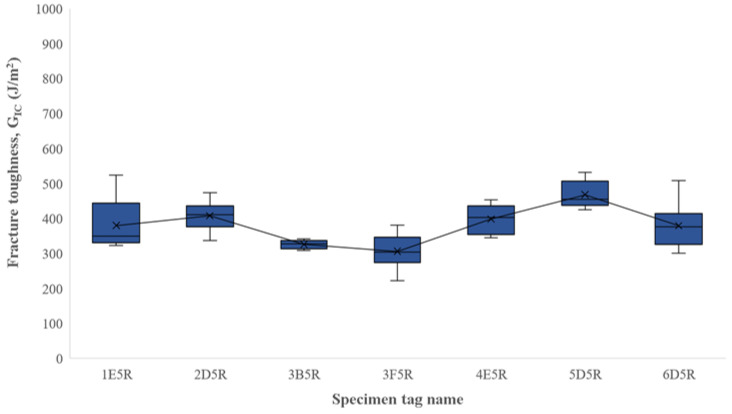
Fracture toughness, G_IC_, for filtered values of specimens using RSCC model.

As mentioned in the explanation for the exploratory work, there were two different types of outlier points. The first type was at the end of the testing produced by catastrophic failure; although common, these outliers were not observed at every testing. As indicated before, the testing method required the test to be stopped before catastrophic failure, but since there was no automated stop protocol, some tests could not be stopped before catastrophic failure. The outliers produced by this failure were filtered by the simple method of just using the peak values at the LvD curve. For the outliers at the beginning of the test (the ones produced using dull pre-crack notch) the filtering was conducted by removing every peak before and at the critical load deflection length, since those loads were also critical values or “peaks”. This was performed by indexing the critical load and creating an array that just included the values after the critical index. As shown in the figure, the peak values of the LvD plots were used to calculate G_IC_ for a single specimen and then paired with their corresponding R-plots, which used the RSCC fracture toughness calculation method (Figure 12). The discrimination described previously was mainly used for removing the outliers. Box plots for the filtered results were also created (Figure 13).

The test was performed at three different deflection speeds of 1, 3, and 5 mm/min, with average G_IC_ values of 377.89, 401.82, and 370.2 J/m^2^ and standard deviations of 65.49, 30.45, and 55.52, respectively. A two-tailed hypothesis *t*-test was performed to the fracture toughness unfiltered values. The *t*-test was conducted to compare the statistical difference of fracture toughness means at different deflection speeds. The hypothesis was that the mean values would be unequal, indicating the deflection speed had a significant impact in fracture calculations. The hypotheses are represented as

$$\;H_{0}:\;{\;\;\;\;\;\mu }_{1}=\mu_{2}\;\;H_{T}:{\;\;\;\;\;\mu }_{1}\neq \mu_{2}$$

where µ_1_ is the mean of a rate, while µ_2_ is the mean of the rate that is being compared to. H_0_ and H_T_ stand for the null hypothesis and the thesis hypothesis, respectively, with a level of significance of α of 0.05. The comparisons made were with the deflection speeds 5/3, 5/1, and 3/1 mm/min; the results of the *t* test were 0.2568, 0.8360, and 0.5858, respectively, for the *p* values. These *p* values are bigger than α, which indicates that the null hypothesis was non-rejectable. Additionally, a one-tailed f test was performed to see if there was any statistical difference variance using, again, an α of 0.05. The hypotheses were as follows:

$$\;H_{0}:\;{\;\;\;\;\;\sigma }_{1}=\sigma_{2}\;\;H_{T}:{\;\;\;\;\;\sigma }_{1}\neq \sigma_{2}$$

where σ_1_ is the variance and σ_2_ is the variance that it is compared to. The H, as before, represents the hypotheses regarding whether there is a statistical difference in variance. The *p* values were 0.1042, 0.3334, and 0.0662, which are still bigger than α, making H_T_ unable to be proven. This finally serves as proof that there is no significant difference in variance or means when calculating fracture toughness across deflection speeds.

It is important to note that, even though the objective of the project was to propose and modernize a reliable testing method for large-scale AM, there was still significant human intervention in the process. Crucial aspects of the testing, such as the application of the coating or the sharpening of the pre-crack, required a user to perform them, which, as mentioned throughout the results, led to inconsistent outcomes. The same can be said for the conditioning method used since, due to the equipment available, it was heavily manual in nature.

The exploratory work showed that due to the testing method showcased in this project, the difference between preload and no-preload is non-existent. The methodology for preload can be upgraded by implementing a better control technique or even implementing real-time image processing for measuring crack propagation to stop the test at the required crack length; however, due to the results of the project, it proves impractical. An argument can be made that a no-preload approach was more practical, as it removed the need to stop the test manually. The exploratory testing also showed that sharpening the pre-crack yielded better characterization of the material properties. This was concluded since specimens with no notch sharpening would sustain higher loads, specifically around 100 N or 10% more loading than the specimens with notch sharpening. Something experienced by specimens with non-sharpened notches was the transition from unstable brittle fracture to stable brittle fracture, while the specimens with a sharpened notch stayed in stable brittle throughout. It was theorized that the cause of the dull notch fracture type could be the load being distributed across the crack tip geometry, meaning a characterization of the structure properties instead of material properties.

The impact of the pre-crack notch varied due to the location of the layer interface in relation to the pre-crack, something that could not be controlled since the water jetting for the coupon harvesting was performed externally. Ideally, the pre-crack notch and interlayer plane should be parallel and as close as possible and measurements should be around these features, including the placement of the loading pins. Testing was randomized, while these measurements were being taken to find any correlation or trend pertaining to these measurements in the future. Some tilt was observed on the specimens as they reached the stability frame. The cause for the tilt was the alignment between the pre-crack notch and the interlayer plane. As mentioned before, the alignment between interlayer and notch was inconsistent due to the specimen fabrication method. This produced a limitation in the testing proposed, since the lack of alignment introduced undesired shear loads that were not compensated for in the calculations. These limitations should be carefully considered. It is important to mention that several specimens were lost because the application of the whiteout coating was not consistent. Some of the specimens did not dry completely, leading to the humid coating covering the crack, causing the program to be unable to measure the crack length. Moving forward, either applying the coating before conditioning or using dry paint would be a worthwhile improvement.

The use of DIC was the main novelty of the project. Although it required additional time to set up, it proved effective in capturing the material behavior. In the future, making the MATLAB code more efficient was identified as a key step for improvement. One way to improve the efficiency was identified to be the automation of the recognition of the calibration dots. The way these calibration dots were identified for this project was by manually setting up two regions of interest (ROIs). A similar ROI set up was required for the detection of crack propagation, marking an opportunity to increase code efficiency.

## 4. Conclusions

In conclusion, the testing method developed in this project was helpful in removing the need for pre-load and reducing human error and DIC allowed for more and more accurate data to be gathered per specimen. Some aspects require further improvement, such as the crack notch sharpening and the application of the white coating. Further automation of the process can be achieved by automating the image calibration process as well as the load data acquisition process. There was no significant statistical difference between using different deflection speeds. Pre-loading did not positively or negatively affect the results gathered by the testing and it is recommended to forego preloading for practicality. Notch sharpening had a significant impact on the results but aligning the notch with the layer interface is more relevant to the success of the test.

## Data Availability

The original contributions presented in the study are included in the article, further inquiries can be directed to the corresponding author/s.
